# Chick paper sampling: a One Health approach to inform public health action during salmonellosis outbreaks linked to backyard poultry in the United States, 2023

**DOI:** 10.3389/fpubh.2025.1705955

**Published:** 2026-01-06

**Authors:** Marta G. Zlotnick, Ashley Aurand-Cravens, Samantha Beaty, Katharine M. Benedict, Karen A. Boegler, Charles R. Clark, Zachary Ellison, Kelly H. Giesbrecht, Samir S. Hanna, Stacy M. Holzbauer, Timothy J. Johnson, Clarissa N. Keisling, Landen Kidd, Carrie A. Klumb, William A. Lanier, Krista Lautenschlager, Melanie Orth, Misha Park Robyn, Grace M. Vahey, Shauna Voss, Kate E. Varela

**Affiliations:** 1 Division of Foodborne, Waterborne, and Environmental Diseases, Centers for Disease Control and Prevention (CDC), Atlanta, GA, United States; 2 Oak Ridge Institute for Science and Education, Oak Ridge, TN, United States; 3 Kentucky Cabinet for Health and Family Services, Frankfort, KY, United States; 4 Tennessee Department of Agriculture, Nashville, TN, United States; 5 Wisconsin Department of Health Services, Madison, WI, United States; 6 ASRT, Inc., Smyrna, GA, United States; 7 Tennessee Department of Health, Nashville, TN, United States; 8 CDC Career Epidemiology Field Officer Program, Centers for Disease Control and Prevention, Atlanta, GA, United States; 9 Minnesota Department of Health, St. Paul, MN, United States; 10 U.S. Public Health Service, Washington, DC, United States; 11 Department of Veterinary and Biomedical Sciences, University of Minnesota, St. Paul, MN, United States; 12 Utah Department of Health and Human Services, Salt Lake City, UT, United States; 13 Utah Department of Agriculture and Food, Ogden, UT, United States; 14 Minnesota Board of Animal Health, Willmar, MN, United States

**Keywords:** chickens, epidemiology, One Health, backyard poultry, salmonella, zoonotic infections

## Abstract

**Introduction:**

Every year, cases of salmonellosis associated with backyard poultry (BYP) contact are reported, despite public health prevention efforts.

**Methods:**

We sampled chick shipping materials (“chick paper”) at agricultural retailers to detect *Salmonella* and to identify supply hatcheries to share mitigation information.

**Results:**

Six states collected 149 samples, and 55 (37%) were positive for *Salmonella*. These yielded 107 *Salmonella* isolates; 47 (44%) of the isolates were *Salmonella* serotypes that matched a subset of the outbreak strains identified in the 2023 multistate BYP-associated outbreak investigation.

**Discussion:**

Isolates obtained through chick shipping material sampling support timely identification of source hatcheries and public health prevention activities. These results suggest chick paper sampling can be a valuable tool during outbreaks linked to BYP.

## Introduction

Backyard poultry (BYP) (chickens, ducks, and other poultry) have increased in popularity in the United States in recent years, with agricultural retail stores reporting record poultry sales during 2020 (1). Each year, human salmonellosis illnesses (infection with *Salmonella* bacteria) associated with BYP contact peak in the spring, coinciding with peak sales of chicks at agricultural stores, despite public health efforts to prevent these illnesses (2). Centers for Disease Control and Prevention (CDC) and state health officials investigate these outbreaks, generally from March through September (3). In 2023, 1,072 BYP-associated salmonellosis (BYPAS) illnesses and 247 hospitalizations were reported in 48 states and Puerto Rico (4). The 2023 BYPAS outbreak comprised 13 *Salmonella* outbreaks (caused by six *Salmonella* serotypes) investigated together due to the common BYP vehicle.

Many BYP owners purchase birds at agricultural retail stores that obtain BYP from hatcheries through distributors or other suppliers (5). Salmonellosis mitigation efforts may take place anywhere along this supply chain (Supplementary material 1). When public health officials suspect that BYP are the source of outbreak patients’ salmonellosis, they attempt to collect samples from a patient’s birds or their home environment to confirm BYP as the outbreak vehicle. Additionally, officials trace patients’ BYP purchases back through the BYP supply chain to identify the source hatchery where the outbreak strain may have originated. If a case of salmonellosis is linked to BYP and traceback identifies a source hatchery, health officials and the hatchery can work together on *Salmonella* monitoring and mitigation to prevent additional illnesses.

Unfortunately, collecting samples to aid an investigation is not always possible. BYP owners, source hatcheries, and other suppliers may be hesitant to allow sampling of birds or their environments. Information available for traceback through patient purchases may be limited by the number of patients willing to complete a supplemental questionnaire specific to backyard poultry contact and by resources at the state level to conduct this extra patient interview. Furthermore, traceback may not converge to a single hatchery because many BYP owners purchase birds from multiple retailers or from retail locations that source their BYP from multiple hatcheries. Thus, there are limits on the amount of data available and the quality of data available for follow-up to identify supplying hatcheries from patient purchase traceback. In the past, hatcheries linked to BYPAS requested to be notified early in the chick season to aid in implementing targeted prevention measures. However, the absence of sampling results and the lack of a clear link between hatcheries and BYP owners had previously delayed or prevented health officials’ communication with hatcheries. Requests for early hatchery notification, coupled with the gap in availability of sampling and traceback data, prompted the need to collect additional laboratory information in the BYP supply chain.

To collect additional laboratory data, chick paper (i.e., material contaminated with poultry fecal material during shipment, such as papers lining shipping boxes, bedding, or box interiors) could be collected from agricultural retail stores (“retailers”) shortly after delivery from a hatchery. If the same *Salmonella* strains were identified on chick paper and in ill people who had contact with BYP, these samples would help confirm BYP as the outbreak vehicle. Hatchery information collected during chick paper sampling would also simplify traceback to the upstream source of *Salmonella* strains causing human illness.

During the 2023 BYPAS outbreak investigation, CDC and state partners collaborated to implement a pilot project, using a One Health approach (6), to collect chick paper samples and hatchery shipping information for traceback from BYP retailers. This pilot project aimed to improve traceback results by informing supply hatcheries of their link to the outbreak and sharing prevention recommendations in a timely manner, with the goal of reducing human illnesses. This report describes the methods and outcomes of this pilot project.

## Materials and methods

CDC epidemiologists lead the multistate response to BYPAS outbreak investigations, including collecting traceback data to inform hatcheries. In 2023, CDC coordinated this pilot project across states at the national level as part of BYPAS outbreak response activities.

### Participant engagement

To identify state partners with potential interest in participating in the pilot project, CDC contacted nine states’ health departments. These health departments successfully collected animal and environmental samples from patients’ BYP that matched the outbreak strains linked to human illness during the 2022 multistate BYPAS outbreak investigation. Engaging in sampling in 2022 provided an indication that these states may have interest and resources for additional sampling in 2023. CDC facilitated individual state calls to describe the project and understand if states had interest and resources to collect chick paper samples during the 2023 BYP outbreak.

### Chick paper sample collection

State partners determined their own sample collection methods, including sampling frequency, timing, location, and coordination with store staff (Table 1). CDC encouraged state partners to begin sampling early in the BYP sales season (approximately March) to link human illness–causing strains to BYP as soon as possible. State partners chose retail stores for sampling based on factors such as geography, personnel capacity, and where ill people reported purchasing poultry during the 2022 multistate BYPAS outbreak. State partners collected information about the shipments (i.e., species and breeds shipped, shipping date, and source hatchery) at the time of sampling. No animals were handled or sampled at the time of chick paper sample collection.

**Table 1:** Sampling and laboratory methods by state.

State	One Health partners	Sampling methods	Lab methods
Kentucky (KY)	KY Cabinet for Health and Family Services KY Department for Public Health Division of Laboratory Services and Division of Epidemiology and Health Planning	State Public Health Veterinarian and interns collected samples. Chick paper, bottom, and sides of boxes were swabbed, targeting obvious soiled areas. Swabs were bagged and taken to the lab right away on ice. Stores visited once a week for 4 weeks.	Chick feces were visible on swabs when they were received at the laboratory. Followed internal standard operating procedure (mod. Bacteriological Analytical Manual [BAM] CH.5) for broth processing. *Pre-Enrichment:* A premoistened swab with the sample was placed in lactose broth. *Enrichment:* Rappaport-Vassiliadis (RV) & Tetrathionate (TT) broths. *Isolation:* Hektoen Enteric (HE), Xylose Lysine Desoxycholate (XLD), and Bismuth Sulfate (BS) agar plates. *Biochemical Screen*: Used on growth: triple sugar iron (TSI) agar, lysine iron (LIA) agar, and urea slants. *PCR:* Confirmation on isolates using the BAX Q7 system.
Minnesota (MN)	MN Department of Health MN Department of Health Public Health Laboratory MN Board of Animal Health University of Minnesota	Store employees were provided with prepared collection kits that included gloves, plastic bags, and written and oral instructions. The store employees placed the chick shipping materials in plastic bags. Collection occurred approximately once a week for 3 weeks.	*Primary processing*: Whole chick papers were immersed in 400 mL of TT broth for primary enrichment for 24 h. *Enrichment*: Primary growths were transferred to RV broth for secondary enrichment. *Isolation*: Cultures were plated onto Xylose-Lysine-Tergitol 4 (XLT-4) agar plates for *Salmonella* isolation. Three colonies were picked from positive plates and submitted for whole-genome sequencing (WGS). Isolates and secondary enrichments were also subjected to *invA* PCR (11).
Montana (MT)	MT Department of Public Health and Human Services MT Public Health Laboratory Flathead County Health Department Sanders County Health Department	Local health department sanitarians established contact with the stores in advance to determine when the chicks would arrive. Swabs of the chick papers were collected by the sanitarians. Swabs were collected from two different stores in one county and from a different store in another county. Each store was sampled on a single date.	Swabs placed in Cary Blair transport media were received at MT Public Health Laboratory. *Primary processing*: MacConkey (MAC) and Hektoen-Enteric (HE) agar plates were streaked with the swabs. *Enrichment*: Gram-negative (GN) broth tubes were inoculated. *Isolation*: The GN broth was plated on additional MAC and HE agar plates. The original plates were held for 48 h, and growth was documented. The GN-sub MAC and GN-sub HE were held for 24 h, and growth was documented.
Tennessee (TN)	TN Department of Health (TDH) TN Department of Agriculture (TDA) Kord Animal Health Diagnostic Lab (KAHDL) TN Department of Health State Public Health Laboratory (TNSPHL)	TN Dept of Agriculture Field Staff, under the State Veterinarian, collected samples after consulting with stores in advance. Chick paper, bottom, and sides of boxes were swabbed, targeting obvious soiled areas using moistened sterile swabs in Ames media. Swabs were packaged on ice and taken directly to or shipped to KAHDL within 24 h. Sample collection occurred once per store.	Initial testing done at KAHDL: *Primary Processing:* Fecal swabs were streaked onto both 5% sheep blood and Hektoen agar plates, then incubated for 24 h at 37 °C. *Enrichment:* Streaked swabs were placed into Selenite broth and left to incubate for 24 h. *Isolation:* Growth on plates was restruck to HE, MAC, and XLT-4 agar plates. Swabs from Selenite broth were struck on Hektoen agar. *Biochemical tests*: Used on growth: Triple Sugar Iron (TSI) agar, Urea slants. *Identification:* All suspected isolates were processed through the MALDI-TOF MS for identification. Positive isolates were serogrouped and sent for PCR confirmation. *PCR Confirmation*: Confirmation on isolates using the 7,500 Fast Realtime PCR system. *Salmonella* colonies, black H2S(+), and also green for chickens were identified via Matrix-assisted laser desorption/ionization time-of-flight mass spectrometry (MALDI-TOF MS). Positive isolates would have been sent to TNSPHL for WGS.
Utah (UT)	UT Department of Health and Human Services (DHHS) and Public Health Lab (UPHL) UT Department of Agriculture and Food (UDAF) UT Veterinary Diagnostic Lab (UVDL)	Public health officials from UT DHHS and a Feed Inspector from UDAF collaborated to collect samples at retail stores. Chick paper was collected directly from chick shipping boxes. The entire chick paper (either the straw mat, chick paper, or both) was collected and bagged in sterile plastic bags. Stores were visited approximately once a week for 4 weeks.	Samples were first cultured at UVDL. *Primary processing*: Whole chick papers were placed in a pre-enrichment broth (peptone) and incubated overnight at 37 °C. *Enrichment*: After overnight incubation, TT broth with brilliant green was prepared and poured into sealed bags containing a portion of the pre-enriched sample and further incubated for 20–24 h at 37 °C. *Isolation*: After incubation, a portion of the enriched culture was transferred to HE and XLT-4 agar plates for *Salmonella* isolation and observed for growth at 37 °C. If *Salmonella* spp. suspect colonies were detected, further testing was performed, including inoculation on selective agar plates and identification using various methods. *WGS*: Isolates were shipped to the UPHL for further WGS analysis.
Wisconsin (WI)	WI Department of Health Services (DHS) La Crosse County Health Department Pierce County Health Department St. Croix County Health Department WI Veterinary Diagnostic Lab (WVDL) WI State Laboratory of Hygiene (WSLH)	Environmental or public health officials collected samples. Individual chick papers were sealed in a large, clean Ziplock bag. Bags were labeled and shipped for testing within 48 h at ambient temperature. Sample collection occurred once per store.	WVDL has a standard operating procedure, following the National Poultry Improvement Program, for testing chick paper samples and regularly accepts samples from poultry producers. *Pre-enrichment*: Samples were pre-enriched in buffered peptone water (BPW). *Enrichment*: Pre-enriched samples were transferred to Tetrathionate-Hajna (TT-Hajna) and Rappaport-Vassiliadis (RV) broths. *Isolation*: Enrichment broths were cultured onto selective agar plates (XLT-4 and Brilliant Green with Novobiocin (BGN)) for *Salmonella* isolation. A total of 3–5 suspect isolates were confirmed by MALDI-TOF/biochemical reactions and then serogrouped and serotyped. Isolates were sequenced by whole-genome multilocus sequence typing (wgMLST) at WSLH.

Following sampling and initial laboratory testing (e.g., broth to plate isolation, culture, and polymerase chain reaction), *Salmonella* isolates were sequenced using whole-genome sequencing (WGS) to determine serotypes (7).

### Linking the chick paper sample results to BYPAS illnesses

State public health laboratories uploaded WGS results from *Salmonella* isolates to the PulseNet national *Salmonella* database (8). PulseNet data analysts searched for additional clinical isolates that were closely genetically related to the chick paper isolates. Chick paper isolates were determined to be either associated with the current BYP outbreak or were considered signals for a new illness outbreak. We defined a chick paper isolate as associated with the current BYP outbreak if it was related within 0–10 allele differences by core genome multi-locus sequence typing (cgMLST) to isolates within an outbreak of human illnesses associated with a *Salmonella* strain undergoing active investigation due to reported BYP exposure among clinical cases. We considered a cluster of chick paper isolates a signal for a new illness outbreak if it was related to a group of 7 or more clinical isolates within 0–10 allele differences by cgMLST, with isolation dates within 60 days of each other (7).

Outbreaks that were the same serotype but were more than 10 alleles different by cgMLST analysis were considered different strains of the same serotype and were classified as separate outbreaks. Each outbreak is numbered to specifically distinguish them by serotype or by outbreak strain of the same serotype.

### Hatcheries and traceback

CDC used information about chick paper source hatcheries to facilitate traceback and hatchery communication for *Salmonella* isolates linked to a BYPAS outbreak.

### Pilot project assessment

After sampling and laboratory testing concluded, CDC conducted semi-structured discussions with project partners to identify facilitators and barriers to sampling and to determine whether chick paper sampling would be feasible during future outbreaks. Key elements and themes were identified.

## Results

### Chick paper sample collection

Six states elected to participate: Kentucky, Minnesota, Montana, Tennessee, Utah, and Wisconsin. Three states (MN, UT, and WI) collected *Salmonella*-positive samples (Table 2). Table 3 lists *Salmonella* serotypes identified and how the samples affected the 2023 BYPAS investigation. Identifying information for isolates from the National Center for Biotechnology Information (NCBI) is available in Supplementary material 2.

**Table 2:** Chick paper sampling quantitative results by state.

State	Number of stores visited	Number of stores yielding positive shipping material samples	Number of samples collected	Number (%) of positive samples	Number of *Salmonella* isolates identified	Number (%) of isolates associated with human illness 2022–2023^*^
Kentucky	2	0	39	0	0	0
Minnesota	6	6	51^**^	42 (82)^**^	94	40 (43)
Montana	2	0	6	0	0	0
Tennessee	4	0	14	0	0	0
Utah	11	6	30	9 (30)	9	5 (56)
Wisconsin	3	2	9	4 (44)	4	2 (50)
Total	28	14	149	55 (37)	107	47 (44)

*Because chick paper samples were acquired and tested early in the 2023 BYPAS investigation, 2022 BYPAS case isolates were used as points of comparison for genetic relatedness of chick paper isolates to Salmonella which had caused human illness. **34/51 samples were culture positive, and isolates were retrieved; 42/51 samples were positive using invA PCR, which included an additional 8/16 culture-negative samples and all culture-positive samples.

**Table 3:** Chick paper sampling serotypes, association with 2023 investigation, and investigation impact.

State	All *Salmonella* serotype(s) isolated	*Salmonella* serotypes associated with 2023 BYP investigation (by outbreak strain ID)*	Investigation impact: Confirmation of known serotype strain outbreak, or identification of an outbreak
Minnesota	Alachua	No association	
	Cerro	No association	
	Enteritidis	Enteritidis outbreak 4	Confirmation of known outbreak
	Infantis	Infantis outbreak 1	Confirmation of known outbreak
	Kentucky	No association	
	Mbandaka	Mbandaka outbreak 1	Confirmation of known outbreak
Utah	Agona	No association	
	Cerro	No association	
	Enteritidis	Enteritidis outbreak 2	Identification and confirmation of the outbreak
	Mbandaka	Mbandaka outbreaks 1 and 2	Confirmation of known outbreak
Wisconsin	Braenderup	Braenderup outbreak 1	Confirmation of known outbreak
	Cerro	No association	
	Enteritidis	Enteritidis outbreak 2	Confirmation of known outbreak
	Kentucky	No association	
Total	8 serotypes	6 outbreak serotypes (47 isolates)	1 outbreak identified and confirmed 5 known outbreaks confirmed

*Six serotypes were included in the 2023 BYPAS outbreak investigation, with smaller genetically related outbreaks within individual serotypes. Outbreaks were defined as being within 0–10 allele differences by cgMLST.

### Linking chick paper sampling results to outbreaks

Samples obtained at 14 of 28 (50%) retailers resulted in the identification of 107 *Salmonella* isolates from 149 samples (72%). PulseNet associated 47 isolates (44%) with human outbreak strains identified during the 2023 BYPAS investigation (7). Of the 13 BYPAS outbreaks identified in the 2023 BYPAS investigation, six (46%) were confirmed to have a BYP vehicle by chick paper sampling results: *Salmonella* Braenderup outbreak 1, *Salmonella* Enteritidis outbreak 2, *Salmonella* Enteritidis outbreak 4, *Salmonella* Infantis outbreak 1, *Salmonella* Mbandaka outbreak 1, and *Salmonella* Mbandaka outbreak 2 (Table 3). One isolate collected in Utah led to the detection of a previously unidentified 2023 outbreak (*Salmonella* Enteritidis outbreak 2).

### Hatcheries and traceback

Of the 47 chick paper samples associated with the 2023 BYPAS outbreak, 40 (85%) were from boxes used to ship chickens, four (9%) were used to ship turkeys, and three (6%) were used to ship ducks. None of these samples came from boxes that shipped multiple species, ruling out the possibility of *Salmonella* cross-contamination from one species to the other during shipping.

Traceback information gathered via chick paper sampling identified four supplying hatcheries (A, B, C, and D) for the six *Salmonella* outbreaks with associated chick paper samples (Table 4). Hatchery A was identified via chick paper sampling, traceback, and traceback information from patient poultry purchases. Hatcheries B, C, and D were identified via chick paper sampling only. One additional hatchery (E) was identified for two outbreaks (*Salmonella* Infantis and *Salmonella* Enteritidis outbreak 2) via traceback from patient purchases and not through chick paper sampling.

**Table 4:** Summary of hatcheries identified by chick paper sampling and outbreak patient purchase traceback by BYPAS outbreak strain, 2023.

Outbreak strain	Hatchery	Identified in chick paper sampling traceback	Identified in outbreak patient purchase traceback
Braenderup 1	Hatchery A	—	Yes
	Hatchery B*	Yes	—
Infantis	Hatchery A	Yes	Yes
	Hatchery E	—	Yes
Mbandaka 1	Hatchery A	Yes	Yes
Mbandaka 2	Hatchery A	Yes	Yes
	Hatchery C*	Yes	—
Enteritidis 2	Hatchery A*	Yes	—
	Hatchery D^*^	Yes	—
	Hatchery E	—	Yes
Enteritidis 4	Hatchery A	Yes	Yes

*Denotes source hatcheries only identified through chick paper sampling for that specific outbreak strain.

### Pilot project assessment

Input from states during participant engagement and after project completion revealed several common themes in facilitators and barriers to sampling (Table 5) and overall benefits of the project to states. Notable common facilitators across states included existing relationships among state and local One Health partners and between these partners and retailers. Participants reported that emphasizing that sampling activities were non-punitive helped to assuage some retailers’ concerns about participating. Insufficient personnel or funding were common barriers cited for states’ inability to participate.

**Table 5:** Selected themes of facilitators and barriers to state partners’ participation in the pilot project and sampling at retail stores during the project.

Themes	Facilitators	Barriers
Health officials’ participation in the project		
Resources and staffing	Funding available for cross-cutting or One Health activities	Insufficient personnel or capacity Insufficient funding, especially for laboratory work
Time		Staff are always busy with outbreaks during spring and summer Insufficient lead time to prepare
Ability/knowledge	Expertise in and process for sampling and testing already present	
Engagement of other partners within the state	Existing good relationships among departments within the state, including laboratories and the Department of Agriculture	
Geography		Large state with decentralized health department(s)
Other priorities	Absence of severe HPAI outbreak in 2023	Conflicting priorities Anticipation that the avian influenza outbreak response would require resources
Other	Previous work on *Salmonella* with CDC	Some feed stores received birds from more than one hatchery
Sampling during the project		
Cooperation and understanding of retail stores	Existing good relationships between state/local officials and retail stores Assured feed stores they would not be punished/implicated State already requires that stores be inspected annually, so store officials knew inspectors	Difficult to contact store staff Store is concerned about liability or penalties if *Salmonella* is found Lack of agreement between the local and corporate retail office on whether to participate
Time	Less time-consuming than sampling at private homes Little extra effort for stores	
Other	Forms, technical assistance provided by CDC	Difficult to arrange sampling soon (e.g., <1 day) after shipment
Both participation in the project and sampling during the project		
Engagement of other partners within the state	Existing relationships between state/local officials	Need engagement from a different department (e.g., agriculture or local health department) that cannot participate
Resources and staffing	Enthusiasm and availability among state/local staff	

Benefits identified by participating state partners included the establishment or reinforcement of local relationships among state officials and local retail stores; specifically, sampling at retail locations allowed for engagement with retailers about zoonotic disease prevention measures and was easier to arrange than sampling at patients’ homes. Additional benefits of sampling included the generation of information about zoonotic disease transmission and the value of preventative measures shared with poultry retailers, as well as the creation of a communication process and workflow for this type of testing if it did not already exist. The project also allowed participating individuals to better see the “whole picture” of the BYP supply chain. Ultimately, the project added to the body of knowledge about *Salmonella* and the risks it poses to humans.

## Discussion

The chick paper sampling pilot project exemplified One Health in action. Human and animal health agencies worked together to facilitate communication with retailers, sample collection, and laboratory testing. Project results advanced the 2023 multistate BYPAS investigation by confirming BYP as the outbreak vehicle, reducing the need for sampling patient-owned BYP, and demonstrating that chick paper sampling in a few states could aid a nationwide investigation.

Chick paper sampling has been used previously to trace BYP to source hatcheries (9, 10); however, this pilot project was the first time chick paper sampling was used to generate confirmatory evidence and stimulate illness prevention efforts during a multistate BYPAS outbreak. Chick paper samples provided supporting evidence for the timely incorporation of *Salmonella* outbreaks into the 2023 BYPAS outbreak data and led to the detection of a previously unrecognized BYPAS outbreak. CDC collaborated with local One Health partners to communicate positive test results linked to the BYPAS outbreak to hatcheries. Both the 2022 and 2023 BYPAS outbreak investigations were initiated in April of the respective year; however, initial hatchery contact took place earlier in the 2023 BYPAS investigation (May and June) than during the 2022 investigation (September). For three BYPAS outbreaks (Braenderup outbreak 1, Mbandaka outbreak 2, and Enteritidis outbreak 2), chick paper sampling traceback identified additional source hatcheries (Hatcheries A, B, C, and D) that would not have been linked to the outbreak through patient purchase traceback alone.

Information generated by chick paper sampling allowed CDC investigators to contact Hatchery A in May 2023 to spur enhanced *Salmonella* mitigation efforts and reduce further *Salmonella* illnesses. While this hatchery was identified in traceback for all six outbreaks with associated chick paper samples, it was also identified for four of the seven other 2023 BYPAS outbreaks; thus, ten of the thirteen 2023 BYPAS outbreaks were linked to this hatchery. Having this additional traceback directly to the hatchery from chick paper samples was valuable for communicating about the overall 2023 BYPAS investigation with the hatchery. Investigators learned that Hatchery A sources some hatching eggs from upstream suppliers; thus, additional information would be needed to determine the *Salmonella* source. Hatchery A also conducts routine *Salmonella* surveillance, but this does not include whole-genome sequencing (WGS). Hatchery A reported that they had not detected outbreaks of *Salmonella* serotypes during their routine surveillance. CDC and state partners engaged with Hatchery A to discuss enhanced surveillance with WGS and possibly incorporating autogenous vaccination of poultry for *Salmonella* serotypes that caused human illnesses in the BYPAS outbreak.

Identification of links to three additional hatcheries (Hatcheries B, C, and D) led to One Health partner engagement with these hatcheries. Representatives from the state Department of Agriculture conducted site visits at Hatchery B and Hatchery D, which included collecting additional samples, reviewing cleaning and disinfection protocols, and providing educational materials to hatchery staff. Hatchery C had not detected *Salmonella* Mbandaka (the serotype of the outbreak linked to them) during routine *Salmonella* surveillance in 2023; however, state partners discussed findings with Hatchery C and learned that Hatchery C was planning to incorporate the *Salmonella* Mbandaka serotype into their vaccine protocol in subsequent breeding seasons based on the findings from chick paper sampling. These *Salmonella* prevention activities and partner engagement would not have happened without the results of chick paper sampling.

One of the source hatcheries, Hatchery D, shipped birds from another hatchery, Hatchery B, to a retailer under Hatchery D’s name. This is a practice called drop-shipping, which usually occurs when a hatchery is unable to fill a customer’s order from its own supply (5). The original hatchery asks a second hatchery to ship poultry directly to the customer under the original hatchery’s name, complicating traceback efforts. Drop-shipping can also lead to mixing birds from multiple hatcheries in one shipment; thus, retail stores and consumers will not know which hatchery their birds originated from or what *Salmonella* monitoring and prevention methods the true source hatchery practices. This highlights the importance of retailers understanding the practices of their source hatcheries.

CDC investigators aimed to better understand whether chick paper sampling should be a recommended sampling modality for state and local One Health partners during future outbreaks. Based on participant input, fostering relationships and trust with retailers should be considered long-term aims for future chick paper sampling, including at the national level for retailers operating in multiple states. For the future success of chick paper sampling, state partners recommended developing uniform sample collection and testing protocols. Partners strongly advocated for dedicated resources for these activities as they proved a foundation and catalyst for future One Health collaborations, including investigating zoonotic infections beyond BYPAS.

The results of this project are subject to several possible limitations. Retailers might not be nationally representative because they were chosen, in part, through convenience sampling. Different sampling and testing methods may have affected laboratory results across participating states. Results of this project indicated that the collection of whole chick papers and immersion of whole chick papers in pre-enrichment broth may have been more successful in isolating *Salmonella* than those samples collected using swabs (Tables 1, 2). A study to validate the laboratory methods used in the project is needed to determine the optimal sample collection and testing protocol. Standardization of the sampling protocol would minimize the impact of collection technique on results and minimize potential cross-contamination of the chick paper after shipment before sampling occurs.

BYPAS remains a complex public health problem requiring partnerships across the One Health sectors. Chick paper sampling proved valuable in efficiently identifying BYPAS during the 2023 multistate investigation, highlighting its potential as a One Health tool for zoonotic disease prevention, surveillance, and investigation across multiple jurisdictions.

## Data Availability

The datasets presented in this study can be found in online repositories. The names of the repository/repositories and accession number(s) can be found in the article/Supplementary material.
