# Supplementary material for: Chick paper sampling: a One Health approach to inform public health action during salmonellosis outbreaks linked to backyard poultry in the United States, 2023
Source: Front Public Health. 2026 Jan 6;13:1705955. doi: 10.3389/fpubh.2025.1705955 (PMC12816334; doi:10.3389/fpubh.2025.1705955)
Supplement: Supplementary file 1 [file Supplementary_file_1.docx]

**SUPPLEMENTS 1-2**

**SUPPLEMENT 1. Figure: Simplified backyard poultry supply chain with potential *Salmonella* transmission interventions.**


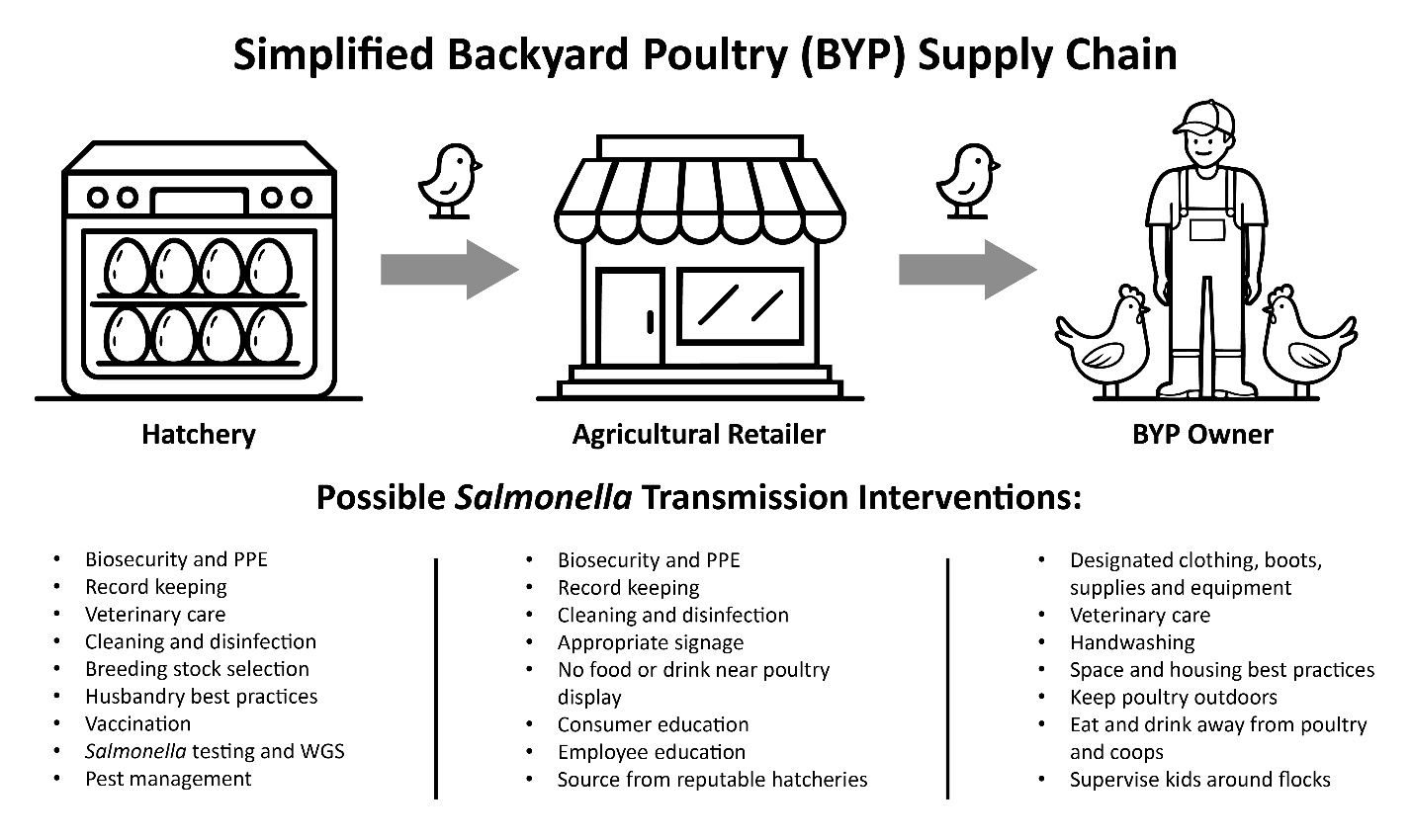


Figure: Simplified backyard poultry supply chain. Backyard poultry typically originate at a hatchery, from which day-old chicks or other baby poultry are shipped to an agricultural retailer for purchase by consumers. Interventions to reduce *Salmonella* transmission can occur at any point along this supply chain. Examples of such interventions include biosecurity and vaccination at the hatchery level, providing consumer educational materials at the retailer level, and hand washing and biosecurity measures at home (1-3). PPE: personal protective equipment. WGS: whole genome sequencing.

**References:**

1. Nichols M, Stevenson L, Whitlock L, Pabilonia K, Robyn M, Basler C, et al. Preventing Human Salmonella Infections Resulting from Live Poultry Contact through Interventions at Retail Stores. J Agric Saf Health. 2018;24(3):155-66.

2. Salmonella Outbreaks Linked to Backyard Poultry: U. S. Centers for Disease Control and Prevention; 2023 [updated October 19, 2023; cited 2023 October 31, 2023]. Available from: <https://www.cdc.gov/salmonella/backyardpoultry-05-23/index.html>.

3. USDA. Best Management Practices Handbook: A Guide to the Mitigation of Salmonella Contamination at Poultry Hatcheries. In: Agriculture USDo, editor. Conyers, GA: USDA-APHIS-VS-National Poultry Improvement Plan; 2014.

**SUPPLEMENT 2 – Identifying information for isolates in the National Center for Biotechnology Information (NCBI).**

| **WGS Identifier** | **BioSample Accession Number** | **NCBI Sequence Run ID** |
| --- | --- | --- |
| PNUSAS343069 | SAMN34194557 | SRR24178948 |
| PNUSAS346797 | SAMN45486856 | SRR31668128 |
| PNUSAS346798 | SAMN45484915 | SRR31668320 |
| PNUSAS349588 | SAMN45486860 | SRR31668148 |
| PNUSAS349589 | SAMN45486849 | SRR31668304 |
| PNUSAS350466 | SAMN35438823 | SRR24773371 |
| PNUSAS350467 | SAMN35438849 | SRR24773389 |
| PNUSAS350468 | SAMN35438839 | SRR24773400 |
| PNUSAS350469 | SAMN35438838 | SRR24773401 |
| PNUSAS350470 | SAMN35438840 | SRR24773399 |
| PNUSAS350471 | SAMN35438817 | SRR24773405 |
| PNUSAS350472 | SAMN35438818 | SRR24773394 |
| PNUSAS350473 | SAMN35438874 | SRR24773333 |
| PNUSAS350474 | SAMN35438875 | SRR24773331 |
| PNUSAS350475 | SAMN35438870 | SRR24773337 |
| PNUSAS350476 | SAMN35438885 | SRR24773381 |
| PNUSAS350477 | SAMN35438905 | SRR24773359 |
| PNUSAS350478 | SAMN35438877 | SRR24773329 |
| PNUSAS350479 | SAMN35438892 | SRR24773374 |
| PNUSAS350480 | SAMN35438878 | SRR24773328 |
| PNUSAS350481 | SAMN35438883 | SRR24773384 |
| PNUSAS350482 | SAMN35438884 | SRR24773383 |
| PNUSAS350483 | SAMN35438906 | SRR24773358 |
| PNUSAS350484 | SAMN35438907 | SRR24773357 |
| PNUSAS350485 | SAMN35438876 | SRR24773330 |
| PNUSAS350586 | SAMN35438893 | SRR24773373 |
| PNUSAS350587 | SAMN35438896 | SRR24773369 |
| PNUSAS350588 | SAMN35438898 | SRR24773367 |
| PNUSAS350589 | SAMN35438894 | SRR24773372 |
| PNUSAS350590 | SAMN35438897 | SRR24773368 |
| PNUSAS350591 | SAMN35438821 | SRR24773332 |
| PNUSAS350592 | SAMN35438834 | SRR24773406 |
| PNUSAS350593 | SAMN35438831 | SRR24773409 |
| PNUSAS350594 | SAMN35438835 | SRR24773404 |
| PNUSAS350595 | SAMN35438832 | SRR24773408 |
| PNUSAS350596 | SAMN35438833 | SRR24773407 |
| PNUSAS350597 | SAMN35438824 | SRR24773360 |
| PNUSAS350598 | SAMN35438830 | SRR24773410 |
| PNUSAS350599 | SAMN35438828 | SRR24773412 |
| PNUSAS350600 | SAMN35438825 | SRR24773415 |
| PNUSAS350628 | SAMN35438826 | SRR24773414 |
| PNUSAS350629 | SAMN35438822 | SRR24773382 |
| PNUSAS350630 | SAMN35438872 | SRR24773335 |
| PNUSAS350631 | SAMN35438871 | SRR24773336 |
| PNUSAS350632 | SAMN35438873 | SRR24773334 |
| PNUSAS350633 | SAMN35438879 | SRR24773327 |
| PNUSAS350634 | SAMN35438829 | SRR24773411 |
| PNUSAS350635 | SAMN35438880 | SRR24773326 |
| PNUSAS350636 | SAMN35438887 | SRR24773379 |
| PNUSAS350637 | SAMN35438827 | SRR24773413 |
| PNUSAS350638 | SAMN35438853 | SRR24773356 |
| PNUSAS350639 | SAMN35438816 | SRR24773416 |
| PNUSAS350640 | SAMN35438815 | SRR24773417 |
| PNUSAS350641 | SAMN35438861 | SRR24773347 |
| PNUSAS350642 | SAMN35438881 | SRR24773325 |
| PNUSAS350643 | SAMN35438888 | SRR24773378 |
| PNUSAS350644 | SAMN35438886 | SRR24773380 |
| PNUSAS350645 | SAMN35438899 | SRR24773366 |
| PNUSAS350646 | SAMN35438900 | SRR24773365 |
| PNUSAS350647 | SAMN35438901 | SRR24773364 |
| PNUSAS350668 | SAMN35438846 | SRR24773392 |
| PNUSAS350669 | SAMN35438845 | SRR24773393 |
| PNUSAS350670 | SAMN35438844 | SRR24773395 |
| PNUSAS350671 | SAMN35438904 | SRR24773361 |
| PNUSAS350672 | SAMN35438903 | SRR24773362 |
| PNUSAS350673 | SAMN35438902 | SRR24773363 |
| PNUSAS350674 | SAMN35438852 | SRR24773386 |
| PNUSAS350675 | SAMN35438889 | SRR24773377 |
| PNUSAS350676 | SAMN35438895 | SRR24773370 |
| PNUSAS350677 | SAMN35438865 | SRR24773342 |
| PNUSAS350678 | SAMN35438866 | SRR24773341 |
| PNUSAS350679 | SAMN35438869 | SRR24773338 |
| PNUSAS350680 | SAMN35438868 | SRR24773339 |
| PNUSAS350681 | SAMN35438862 | SRR24773346 |
| PNUSAS350682 | SAMN35438882 | SRR24773385 |
| PNUSAS350683 | SAMN35438863 | SRR24773345 |
| PNUSAS350684 | SAMN35438867 | SRR24773340 |
| PNUSAS350685 | SAMN35438859 | SRR24773349 |
| PNUSAS350686 | SAMN35438860 | SRR24773348 |
| PNUSAS350687 | SAMN35438856 | SRR24773352 |
| PNUSAS350708 | SAMN35438857 | SRR24773351 |
| PNUSAS350709 | SAMN35438858 | SRR24773350 |
| PNUSAS350710 | SAMN35438854 | SRR24773355 |
| PNUSAS350711 | SAMN35438855 | SRR24773353 |
| PNUSAS350712 | SAMN35438890 | SRR24773376 |
| PNUSAS350713 | SAMN35438891 | SRR24773375 |
| PNUSAS350714 | SAMN35438864 | SRR24773344 |
| PNUSAS350715 | SAMN35438847 | SRR24773391 |
| PNUSAS350716 | SAMN35438851 | SRR24773387 |
| PNUSAS350717 | SAMN35438850 | SRR24773388 |
| PNUSAS350718 | SAMN35438820 | SRR24773343 |
| PNUSAS350719 | SAMN35438837 | SRR24773402 |
| PNUSAS350720 | SAMN35438843 | SRR24773396 |
| PNUSAS350721 | SAMN35438842 | SRR24773397 |
| PNUSAS350722 | SAMN35438841 | SRR24773398 |
| PNUSAS350723 | SAMN35438819 | SRR24773354 |
| PNUSAS350724 | SAMN35438848 | SRR24773390 |
| PNUSAS350725 | SAMN35438836 | SRR24773403 |
| PNUSAS351056 | SAMN35348846 | SRR24727108 |
| PNUSAS351057 | SAMN35348863 | SRR24727106 |
| PNUSAS351058 | SAMN35348851 | SRR24727084 |
| PNUSAS351059 | SAMN35348444 | SRR24727069 |
| PNUSAS351060 | SAMN35348858 | SRR24727105 |
| PNUSAS351061 | SAMN35348854 | SRR24727086 |
| PNUSAS351062 | SAMN35348856 | SRR24727159 |
| PNUSAS351063 | SAMN35348866 | SRR24727107 |
| PNUSAS354716 | SAMN35684951 | SRR24883016 |
